# Equine Endometrosis Pathological Features: Are They Dependent on NF-κB Signaling Pathway?

**DOI:** 10.3390/ani11113151

**Published:** 2021-11-04

**Authors:** Tomasz Jasiński, Łukasz Zdrojkowski, Ewa Kautz, Edyta Juszczuk-Kubiak, Graça Ferreira-Dias, Małgorzata Domino

**Affiliations:** 1 Department of Large Animal Diseases and Clinic, Institute of Veterinary Medicine, Warsaw University of Life Sciences, 02-787 Warsaw, Poland; tomasz_jasinski@sggw.edu.pl; 2 Department of Molecular Biology, Institute of Genetics and Animal Biotechnology of the Polish Academy of Sciences, 05-552 Jastrzębiec, Poland; e.kautz@igbzpan.pl; 3 Laboratory of Biotechnology and Molecular Engineering, Department of Microbiology, Prof. Wacław Dąbrowski Institute of Agricultural and Food Biotechnology—State Research Institute, 02-532 Warsaw, Poland; edyta.juszczuk-kubiak@ibprs.pl; 4 Departmento de Morfologia e Função, CIISA—Centro de Investigação Interdisciplinar em Sanidade Animal, Faculdade de Medicina Veterinária, Universidade de Lisboa, 1300-47 Lisbon, Portugal; gmlfdias@fmv.ulisboa.pt

**Keywords:** endometrosis, mare, NF-κB, MCP-1, IL-6, HAS

## Abstract

**Simple Summary:**

Endometrosis is a serious problem mainly affecting older mares’ fertility. Despite the importance of this disease, its etiology and pathogenesis are not fully known. Thus, no effective treatment exists to cease or restore degenerative processes and fibrogenesis in the mares’ endometria. The nuclear factor kappaB (NF-κB) is an important factor regulating cell metabolism. Nevertheless, it is also known to promote inflammation and fibrosis in various tissues and species, as well as in the mares’ endometria. The main goal was to bring new knowledge regarding endometrosis pathogenesis, which could allow for therapy development. Endometrial samples, collected postmortem from cyclic mares in estrus or diestrus, were classified histologically and used for gene expression assessment. Gene transcription of NF-κB subunits (subunit RelA—*RelA*; subunit 1—*NF-κB1*; subunit 2—*NF-κB2*), pro-inflammatory molecules (monocyte chemoattractant protein-1—*MCP-1*; interleukin-6—*IL-6*), and hyaluronan synthases (hyaluronan synthase 1—*HAS 1*; hyaluronan synthase 2—*HAS 2*; hyaluronan synthase 3—*HAS 3*) were compared among endometrosis types (active, non-active, destructive, non-destructive), according to the classification of Hoffmann and co-authors. These results suggest that activation of the NF-κB canonical pathway is involved especially in destructive endometrosis, the type when endometrial glands are damaged. These data give substantial information for further evaluations and treatment development.

**Abstract:**

Endometrosis is an important mares’ disease which considerably decreases their fertility. As classic endometrial classification methods might be insufficient for tissue pathological evaluation, further categorization into active/inactive and destructive/non-destructive types was developed by Hoffmann and others. This study aimed to compare NF-κB pathway genes transcription among histopathological types of endometrosis, following Hoffmann and co-authors’ classification. Endometrial samples, collected postmortem from cyclic mares (*n* = 100) in estrus or diestrus, were classified histologically and used for gene transcription assessment. Gene transcription of NF-κB subunits (*RelA*, *NF-κB1*, *NF-κB2*), pro-inflammatory molecules (*MCP-1*, *IL-6*), and hyaluronan synthases (*HAS 1*, *HAS 2*, *HAS 3*) was compared among endometrosis types (active, non-active, destructive, non-destructive). Most individual mRNA samples showed high expression of *RelA*, *NF-κB1*, and *MCP-1* gene transcripts and the destructive type of endometrosis, simultaneously. The expression of *RelA* and *NF-κB1* genes was higher in active destructive group than in the other groups only in the follicular phase, as well as being higher in the inactive destructive group than in the others, only in the mid-luteal phase. The increase in gene transcription of the NF-κB canonical activation pathway in destructive endometrosis may suggest the highest changes in extracellular matrix deposition. Moreover, the estrous cycle phase might influence fibrosis pathogenesis.

## 1. Introduction

Degenerative Endometrial Fibrosis, also referred to as endometrosis, is a major problem in equine reproduction, negatively affecting mares’ fertility. As its occurrence is widespread, endometrosis is an important cause of financial losses in the equine breeding industry. Main paramount features of this disease encompass periglandular fibrosis of the endometrium and degenerative changes of endometrial glands associated with dysfunction of affected glandular epithelial cells [1,2]. Since adequate endometrial gland response is crucial in the nutrition of the embryo, as the severity of endometrosis increases, the risk of embryonic death increases [1,3]. The basic classification method, introduced by Kenney and Doig (1986), focuses on the percentage of affected glands and layers of periglandular fibrosis. This classification allocates uterine biopsy samples into categories I, IIA, IIB, and III [3]. However, more recent studies have assessed the damage of glandular epithelial cells and the metabolic activity of periglandular stromal cells as the basis for the development of an additional endometrosis classification system into four histopathological types [1,2,4]. This classification divides uterine biopsy samples using the terms destructive or nondestructive endometrosis for the description of the damaging glands, and active or inactive endometrosis for the characterization of the metabolic activity of the stroma. Following Schöniger and Schoon’s opinion, these two classifications complement each other, and their combination allows for a better description of the affected mare’s endometrium [2]. It should be kept in mind that the better the description of the current state of the endometrium, the more adequate the assessment of the severity of the endometrial fibrosis, and hence a more accurate prognosis of the future fertility of the mare [1,2,4].

Even though these two classification methods were developed, the etiology and pathogenesis of endometrosis still require further clarification. Some investigation has been carried out on cellular differentiation and periglandular myofibroblast transformation [1,5,6,7], the cycle of asynchronous differentiation [1,4,8,9], failure of innate immunity [10,11,12], extracellular matrix (ECM) composition [13,14,15], and the role of proinflammatory molecules and neutrophil extracellular traps action in stroma fibrosis remodeling [16,17,18,19]. Therefore, numerous biological indicators of endometrial pathophysiology are in the field of interest as biomarkers that can be objectively evaluated during prognostic or diagnostic protocols and treatment responses. Among such biomarkers of endometrosis, the proteins calponin, vimentin, desmin, and smooth muscle actin [1,5,6,7] have been proposed for the assessment of myofibroblast transformation. In addition, estrogen and progesterone receptors, uteroglobin, uterocalin, calbindin, and glycogen [1,4,8,9] have been used as indicators of cycle asynchronous differentiation. Likewise, ß-defensin and indoleamine 2,3-dioxygenase 1 have been suggested as indicators of innate immunity failure in the cytoplasm of endometrosis affected glands [10,11,12]. Moreover, hyaluronan synthases (HASs) have been suggested as the indicators of the production of ECM components with diverse biological functions [13,14,15], as well as monocyte chemoattractant protein-1 (MCP-1), interleukin-6 (IL-6), and tumor necrosis factor α (TNFα) as the proinflammatory molecules involved in the activation of fibrogenesis pathway by acting on cells residing in ECM [16,17,18,19,20]. Given the need to evaluate regular and abnormal cellular function within the mares’ endometria, some of these biomarkers should be considered to have prognostic value for breeding success or for the response to treatment of fibrosis [2,21].

It is worth noting that in humans, the inhibition of the nuclear factor kappaB (NF-κB) pathway is one of the most popular research approaches for the prevention and treatment of fibrosis-related diseases [22,23,24,25,26]. The NF-κB is a pleiotropic transcriptional regulator of the transcription of genes that are responsible for immunity and inflammatory functions [27]. The NF-κB family of proteins consists of c-Rel, RelA (p65), RelB, NF-κB1 (p50/p105), and NF-κB2 (p52/p100) [27,28], which are systematized into two activation pathways—canonical (RelA, NF-κB1) and non-canonical (RelB, NF-κB2) [28,29]. Both pathways lead to the degradation of the inhibitory protein IκBα or C-end of p100, respectively [28,29]. This proteasome-dependent degradation is responsible for the release of NF-κB to the nucleus, causing numerous gene expression initialization, e.g., MCP-1, IL-6, and HAS [23,27,28]. Interestingly, in our recent research, the endometria of healthy mares were mostly devoid of NF-κB pathway gene expression, whereas those with endometrosis frequently showed high expression of RelA in category III endometria, high expression of NF-κB1 in categories IIA, IIB, and III, as well as high expression of NF-κB2 in categories IIA and III. These differences have been predominantly shown in the follicular phase of the estrous cycle [20]. Since delivered results provided interesting insights for NF-κB signaling pathway involvement in endometrosis pathogenesis regarding basic Kenney and Doig classification [20], further studies are required for better understanding the relationship between histopathological features of affected mares’ endometria and the NF-κB signaling pathway, using the endometrosis classification of Hoffmann and co-authors [1]. Thus, this study aimed to evaluate the expression of genes involved in the NF-κB signaling pathway in mares’ endometria in relation to Hoffmann et al. [1] four histopathological types of equine endometrosis. Specifically, gene transcription of NF-κB subunits (*RelA*; *NF-κB1*; *NF-κB2*), pro-inflammatory molecules (*MCP-1*; *IL-6*) and hyaluronan synthases (*HAS 1*; *HAS 2*; *HAS 3*) was compared among four endometrosis types (active, non-active, destructive, non-destructive) at different phases of the estrous cycle (estrus, diestrus). In the future, it is expected, as an ultimate goal, to further evaluate the potential use of NF-κB inhibitors in prophylaxis and treatment of equine endometrosis.

## 2. Materials and Methods

### 2.1. Sample Collection

Biological material for this study consisted of equine internal genitalia and blood collected from 100 Polish warmblood mares (aged from 4 to 25 years). At a commercial slaughterhouse in Poland, samples were collected postmortem in the reproductive season (from April to September), following the European (Council Regulation (EC) No 1099/2009) and Polish (Regulation (MARD) Dz.U. 2004 205 poz. 2102) welfare mandates. No permission from the Ethical Committee following the National Legal Regulation (Act of 15 January 2015 on the Protection of Animals Used for Scientific or Educational Purposes, Dz.U. 2015 poz. 266) was needed for sample collection after slaughter.

Blood samples, each with a volume of 10 mL, were collected during exsanguination into dry tubes for hormone concentration analyses (BD Vacutainer^®^, Plymouth, UK). Blood samples were transported to the laboratory at +4 °C, and centrifuged (2000× *g*, 5 min), for serum retrieval, kept at −20 °C until further hormonal analysis. Serum progesterone (P4) concentration was determined using a commercial radioimmunoassay with the sensitivity of 0.15 ng/mL (KIP 1458; DIAsource ImmunoAssays SA, Ottignies-Louvain-la-Neuve, Belgium; intra-assay coefficient of variation <5.6%; inter-assay coefficient of variation <8.8%). The sample dilution recommended by the manufacturer’s protocol was used. The absorbance was measured by Multiscan Reader (Labsystem, Helsinki, Finland) using Genesis V 3.00 software.

Ovaries were collected into containers with cold saline (0.9% NaCl, Polfa S.A., Lublin, Poland) for macroscopic examination, transported at +4 °C to the laboratory, and sectioned. The presence of follicles and/or corpus luteum was noted, and their diameter was measured.

From each animal, two endometrial samples from the uterine body were collected immediately after evisceration, no longer than 5 min after the mare’s death by exsanguination. One sample was inserted into containers with the 10% neutral phosphate-buffered formalin (Sigma-Aldrich, Poznan, Poland) for histological examination, and the second one into RNase-free Eppendorf tubes (Eppendorf AG, Hamburg, Germany), snap-frozen in liquid nitrogen, and stored at −80 °C for gene transcription analyses. Endometrial samples for histopathological examination were fixed in formalin for 24 h and then moved to 70% ethanol (Sigma-Aldrich, Poznan, Poland) for one week, and then processed for paraffin-embedded blocks.

### 2.2. Phases of Estrous Cycle Determination

The phases of the estrous cycle were determined based on the P4 concentration, and on the macroscopic examination of mares’ ovaries, following Roberto da Costa et al. protocol [30]. Mares were included in the mid-luteal phase group (MLP) when serum P4 concentration was >1 ng/mL and on both ovaries, none of the follicles were >35 mm in diameter, and at least one corpus luteum was demonstrated. Mares were assigned into the follicular phase group (FLP) when serum P4 concentration was <1 ng/mL, and there was at least one follicle >35 mm in diameter in any of the ovaries, and no corpus luteum present. Each group, MLP and FLP, consisted of 50 mares (total *n* = 100). None of the mares were excluded due to the failure of inclusion into any one of the two phases of the estrous cycle.

### 2.3. Histopathological Types of Endometrosis

Fixed endometrial samples were embedded in paraffin equivalent for standard histological staining procedures. The paraffin blocks were cut in 9 μm sections on rotation microtome Leica RM2255 (Kawa-Ska, Zalesie Gorne, Polska) and mounted on glass slides. Then, slides were deparaffinized and rehydrated in a series of immersions in xylene and decreasing concentrations of ethanol (Sigma-Aldrich, Poznan, Poland). Samples were stained using standard hematoxylin-eosin (HE) protocol (hematoxylin, 3801520E, Leica, Buffalo Grove, IL, United States; eosin, HT1103128; SigmaAldrich, Poznan, Poland) and mounted under Canadian balsam resin for histological evaluation (Sigma-Aldrich, Poznan, Poland).

The HE-stained slides were evaluated under a light microscope (Olympus BX43, Warsaw, Poland, magnification 40×–1000×) to assess the presence of inflammation and the appearance or severity of pathological degenerative changes. Endometrial samples that were chosen for RNA isolation did not appear actively inflamed in the macroscopic examination and did not reveal any inflammatory cell infiltration in the histopathological examination. Endometrosis was recognized when the microscopic hallmark, the concentric arrangement of stromal cells and/or collagen fibers around affected glands, was observed [2,31]. Endometrial samples were classified as belonging to histopathological types inactive nondestructive (IN), inactive destructive (ID), active nondestructive (AN), and active destructive (AD), according to specific pathological features [1,2]. In the nondestructive type, glandular epithelial cells were intact, whereas in the destructive type of endometrosis, degenerative lesions and necrosis were observed. The features of periglandular stromal cells’ metabolic activity allowed for the inclusion into the active or inactive type. In the active type, active stromal cells characterized by an oval shape, pale cytoplasm, and ovoid hypochromatic nuclei were noted, whereas in the inactive type, inactive stromal cells with spindle-shaped elongated hyperchromatic nuclei were observed [2].

**Figure 1:**
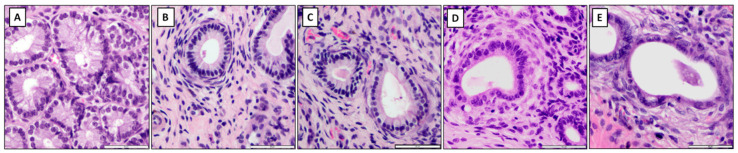
The microscopic image of exemplary endometrial sections classified into the following groups: (**A**) the control group, (**B**) the inactive nondestructive endometrosis, (**C**) the inactive destructive endometrosis, (**D**) the active nondestructive endometrosis, (**E**) the active destructive endometrosis. HE staining. magnification 400×.

The mares with healthy endometrial tissue were included in the control group (C; *n* = 20). In addition, the remaining 80 mares were assigned to each of the four endometrosis histopathological types, as follows: (i) inactive nondestructive endometrosis (E IN; *n* = 20), (ii) inactive destructive endometrosis (E ID; *n* = 20), (iii) active nondestructive endometrosis (E AN; *n* = 20), and (iv) active destructive endometrosis (E AD; *n* = 20) (Figure 1). In all groups, half of the samples were collected from mares in FLP (C, *n* = 10; E IN, *n* = 10; E ID, *n* = 10; E AN, *n* = 10; E AD, *n* = 10) and the other half in MLP (C, *n* = 10; E IN, *n* = 10; E ID, *n* = 10; E AN, *n* = 10; E AD, *n* = 10). Part of the results on the transcription of selected genes involved in the NF-kB signaling pathway in endometria were classified in Kenney and Doig’s (1986) categories, I, IIA, IIB, III, as previously documented [20].

### 2.4. Gene Transcription Evaluation

Frozen endometrial samples were mechanically disrupted in a liquid nitrogen environment. Afterwards, 50 mg of each sample were homogenized in Lysing Matrix D tubes (MP Biomedicals, Irvine, CA, United States), and total RNA was extracted using High Pure RNA Tissue Kit (Roche, Rotkreuz, Switzerland). The extraction protocol recommended by the manufacturers was used. Then, a DNase treatment was performed. The RNA concentration was determined using DS-11 FX spectrophotometer (DeNovix, Wilmington, DE, United States) with absorbance ratios A260/280 and A260/230 of approximately 2.0. The further analysis inclusion criterion was RNA concentration above 100 ng. None of the samples were excluded due to insufficient RNA concentration.

Real-time PCR (qPCR) amplification was performed using a TaqMan™ RNAto-CT™ 1-Step Kit (No 4392938, ThermoFisher, Swedesboro, NJ, United States) and a Quant-Studio™ 6 Flex Real-Time PCR System (Applied Biosystems, Wilmington, DE, United States). The commercially available equine-specific TaqMan Gene Expression Assays (No 4448892 and 4441114, ThermoFisher, Swedesboro, NJ, United States) were used. The list of primers and 6-carboxyfluorescein (6-FAM) and 6-carboxytetramethylrhodamine (TAMRA)-labeled TaqMan probes used for the qPCR analysis was presented in Domino et al. [20]. Real-time PCR reaction had a 10 mL volume and included 15 ng of total RNA, 5 mL of TaqMan^®^ RTPCR Mix (2×), 0.25 mL of TaqMan^®^ RT Enzyme Mix (40×), 0.5 mL of TaqMan probe, and both PCR primers (ThermoFisher, Swedesboro, NJ, USA) for each gene of interest. The PCR protocol included four steps as follow reverse transcription (15 min at 48 °C), enzyme activation (10 min at 95 °C), 40 cycles of denaturation (15 s at 95 °C) and annealing/extension (1 min at 60 °C). Each sample was run in triplicate.

### 2.5. Data Analysis

Each endometrial sample was double categorized using estrous cycle criterion and endometrosis criterion. The estrous cycle determination data were listed as MLP or FLP for each endometrial sample. Independently, healthy endometria (C), and histopathological types of endometrosis data were allocated to each one of the following groups: C, E IN, E ID, E AN, or E AD. In each endometrial sample, transcription of the following eight genes was assessed by qPCR: *RelA*, *NF-κB1*, *NF-κB2*, *MCP-1*, *IL-6*, *HAS 1*, *HAS 2*, and *HAS 3*. Raw data of genes transcription were normalized using the geometric mean of mRNA detected from two independent endogenous reference genes (*GAPDH*, *HPRT1*). The semi-quantitation of the target gene expression was performed in a comparative CT method (ΔΔCT method), where the target gene expression in the samples of category C was considered as ΔCt Control Value.

### 2.6. Statistical Analysis

Univariate marginal distributions of Expression Fold Change (2^−ΔΔCt^) of the qPCR data were tested independently for each endometrial samples category and each target gene using a univariate Kolmogorov–Smirnov test. The comparison between histopathological types was assessed by Kruskal–Wallis test, followed by Dunn’s multiple comparisons test. The comparison between phases of estrous cycle was performed Unpaired t-test with Welch’s correction for normally distributed data pairs or Mann–Whitney test for non-Gaussian data pairs. Numerical data were reported on the box plots using minimum and maximum values, lower and upper quartiles, as well as median. The control group level was estimated as a maximal value of gene expression in a control group and marked on plots using a dashed line. The destructive group level was introduced when only destructive type samples were above the marked level. The destructive group level was marked on selected plots using a dashed line. The percentages of samples in each histopathological type with the value above the level of control group and destructive type were also calculated. All statistical analysis was performed using GraphPad Prism6 software (GraphPad Software Inc., San Diego, CA, USA), where the significance level was established as *p* < 0.05.

## 3. Results

**Figure 2:**
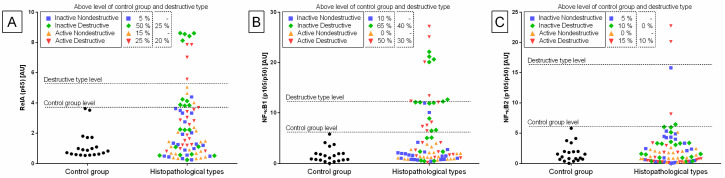
Distribution of (**A**) the gene transcription of the nuclear factor-κB subunit RelA (*RelA*; *p65*), (**B**) nuclear factor-κB subunit 1 (*NF-κB1*; *p105/p50*), and (**C**) nuclear factor-κB subunit 2 (*NF-κB2*; *p100/p52*) in the mares’ endometria in control group, and histopathological types marked with: blue squares, for the inactive nondestructive endometrosis (*n* = 20); green diamonds, for the inactive destructive endometrosis (*n* = 20); orange triangles, for the active nondestructive endometrosis (*n* = 20); and red triangles, for the active destructive endometrosis (*n* = 20). The percentage of samples that showed transcript values of a specific gene above the control group level and destructive type level are indicated in the dashed rectangles.

**Figure 3:**
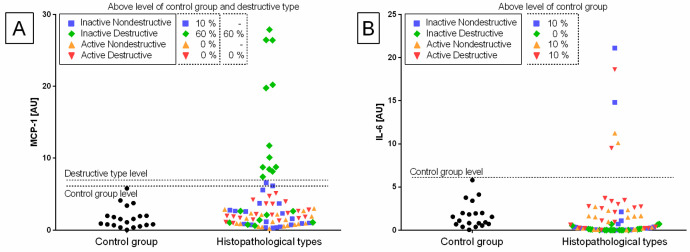
Distribution of (**A**) the gene transcription of monocyte chemoattractant protein-1 (*MCP-1*), and (**B**) interleukin-6 (*IL-6*) in the mares’ endometria in control group and histopathological types marked with blue squares for the inactive nondestructive endometrosis (*n* = 20); green diamonds, for the inactive destructive endometrosis (*n* = 20); orange triangles, for the active nondestructive endometrosis (*n* = 20); and red triangles, for the active destructive endometrosis (*n* = 20). The percentage of samples that showed transcript values of a specific gene above the control group level and destructive type level are indicated in the dashed rectangles.

**Figure 4:**
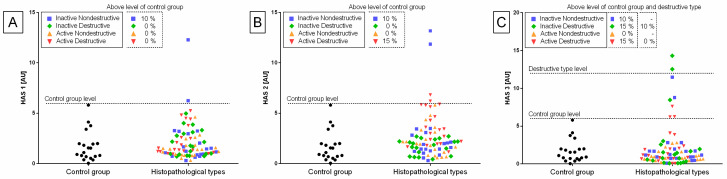
Distribution of gene transcription of (**A**) hyaluronan synthase 1 (*HAS 1*), (**B**) hyaluronan synthase 2 (*HAS 2*), and (**C**) hyaluronan synthase 3 (*HAS 3*) in the mares’ endometria in control group and histopathological types marked with blue squares, for the inactive nondestructive endometrosis (*n* = 20); green diamonds, for the inactive destructive (*n* = 20); orange triangle, for the active nondestructive endometrosis (*n* = 20); and red triangles, for the active destructive endometrosis (*n* = 20). The percentage of samples that showed transcript values of a specific gene above the control group level and destructive type level are indicated in the dashed rectangles.

The heterogeneity of the distribution of transcripts of target genes in the control and endometrosis groups was observed (Figure 2, Figure 3 and Figure 4), hence the intra-group variability was high. Therefore, the individual samples distribution was visualized including individuals of each endometrosis histopathological type and healthy endometrium.

Individual sample distribution of mRNA levels of NF-κB subunits (Figure 2), proinflammatory proteins (Figure 3), and hyaluronan synthases (Figure 4) differed in relation to the control group. Most individual transcript samples above the level of the control group represented *NF-κB1* gene (60% E ID, 50% E AD, and 10% E IN), then *RelA* gene (50% E ID, 25% E AD, 15% E ID, and 5% E IN), *MCP-1* gene (60% E ID and 10% E IN), and *NF-κB2* gene (15% E AD, 10% E ID, and 5% E IN). For these four target genes, the individual distribution of transcripts of the destructive type dominated over the non-destructive type.

Transcription levels of all the genes under study were calculated for the destructive type. Most individual mRNA samples above the level of the destructive type corresponded to *MCP-1* gene transcripts (60% E ID), *NF-κB1* gene transcripts (40% E ID and 30% E AD), and *RelA* transcripts (25% E ID and 20% E AD). However, transcription levels of *NF-κB2* and *HAS 3* above the level of the destructive type of endometrium only occurred in 10% of E AD for *NF-κB2* and 10% of E ID for *HAS 3* endometria. The remaining target genes transcripts did not meet the destructive type-level criterion.

**Figure 5:**
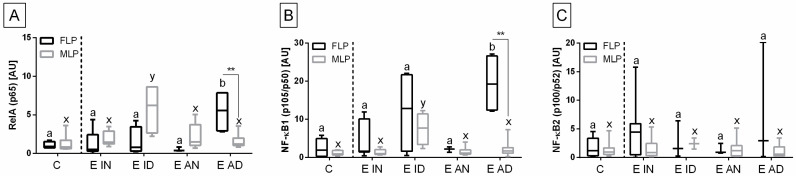
The transcription levels (minimum value, lower quartile, median, upper quartile, and maximum values) of (**A**) the nuclear factor-κB subunit RelA (*RelA; p65*), (**B**) nuclear factor-κB subunit 1 (*NF-κB1*; *p105/p50*), and (**C**) nuclear factor-κB subunit 2 (*NF-κB2*; *p100/p52*) in the mare’s endometria in follicular (FLP; *n* = 50) or mid-luteal (MLP; *n* = 50) phases of the estrous cycle. The endometrial samples classified as inactive nondestructive endometrosis (E IN; *n* = 20), inactive destructive endometrosis (E ID; *n* = 20), active nondestructive endometrosis (E AN; *n* = 20), or active destructive endometrosis (E AD; *n* = 20), and compared with the control group (C; *n* = 20). Lower case letters indicate differences between histopathological types of endometrosis for *p* < 0.05. Asterisks indicate differences between phases of estrous cycle (** *p* < 0.01).

**Figure 6:**
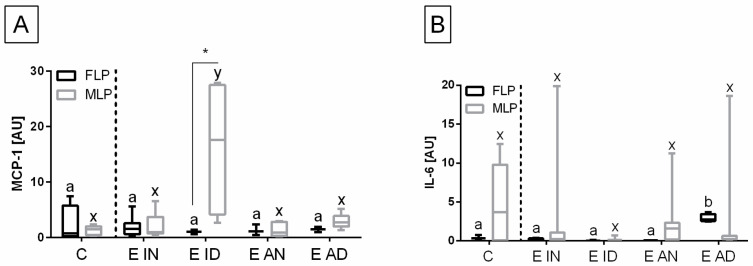
The transcription levels (minimum value, lower quartile, median, upper quartile, and maximum values) of (**A**) monocyte chemoattractant protein-1 (*MCP-1*) and (**B**) interleukin-6 (*IL-6*) in the mares’ endometria in follicular (FLP; *n* = 50) or mid-luteal (MLP; *n* = 50) phases of the estrous cycle. The endometrial samples classified as inactive nondestructive endometrosis (E IN; *n* = 20), inactive destructive endometrosis (E ID; *n* = 20), active nondestructive endometrosis (E AN; *n* = 20), or active destructive endometrosis (E AD; *n* = 20), and compared with the control group (C; *n* = 20). Lower case letters indicate differences between histopathological types of endometrosis for *p* < 0.05. Asterisks indicate differences between phases of estrous cycle (* *p* < 0.05).

**Figure 7:**
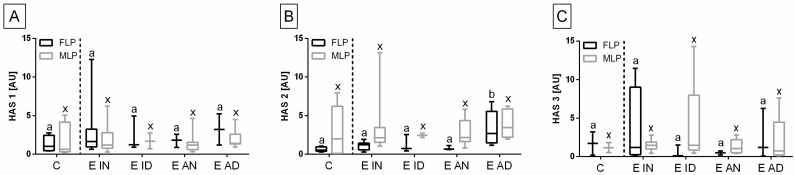
The expression levels (minimum value, lower quartile, median, upper quartile, and maximum values) of (**A**) hyaluronan synthase 1 (*HAS 1*), (**B**) hyaluronan synthase 2 (*HAS 2*), and (**C**) hyaluronan synthase 3 (*HAS 3*) in the mares’ endometria in follicular (FLP; *n* = 50) and mid-luteal (MLP; *n* = 50) phases of the estrous cycle. The endometrial samples classified as inactive nondestructive endometrosis (E IN; *n* = 20), inactive destructive endometrosis (E ID; *n* = 20), active nondestructive endometrosis (E AN; *n* = 20), or active destructive endometrosis (E AD; *n* = 20), and compared with the control group (C; *n* = 20). Lower case letters indicate differences between histopathological types of endometrosis for *p* < 0.05.

The transcription of the genes under study in the histopathological types of equine endometrosis differed in relation to the control group for NF-κB subunits of canonical (*RelA*, *NF-κB1*), but not for the non-canonical (*NF-κB2*) pathway (Figure 5), for *MCP-1* and *IL-6* (Figure 6), as well as for *HAS 2*, but not for *HAS 1* and *HAS 3* (Figure 7).

The transcript expression of *RelA* gene was higher in E AD (*p* = 0.033) than in the other groups in the follicular phase, but not in the mid-luteal phase of the estrous cycle, as well as in E ID (*p* = 0.018) in mid-luteal phase, but not in the follicular phase. Moreover, *RelA* gene transcription also differed between follicular and mid-luteal phases in the E AD (*p* = 0.006; *RelA* higher in FLP), but not in E ID (*p* = 0.114) histopathological types (Figure 5A). Similarly, the transcription of *NF-κB1* gene increased more in E AD (*p* = 0.044) than in the other groups in the follicular phase, but not in the mid-luteal phase. In addition, similarly to *RelA*, the transcription of *NF-κB1* gene was higher in E ID (*p* = 0.023) than in the other groups in mid-luteal phase, but not in the follicular phase. The *NF-κB1* gene transcription was raised in the follicular phase, only in the E AD (*p* = 0.004) histopathological type (Figure 5B). No differences in the expression of *NF-κB2* gene were found between either the histopathological type or phases of the estrous cycle (Figure 5C).

The transcription of the *MCP-1* gene in the endometrium was higher in E ID (*p* = 0.030), than in the other groups, but only in the mid-luteal phase. In addition, *MCP-1* gene transcript was higher in the mid-luteal than in the follicular phases in the E ID histopathological type (*p* = 0.047) (Figure 6A). On the contrary, the transcription of *IL-6* gene was increased in E AD (*p* = 0.028) than in the other groups, but just in the follicular phase. However, no differences in the transcript level of *IL-6* were found between phases of estrous cycle in E AD (*p* = 0.343), and in any other histopathological groups (Figure 6B).

No differences in the transcription of *HAS 1* (Figure 7A) and *HAS 3* (Figure 7C) genes were found between either histopathological type of endometrosis or phases of estrous cycle. However, the transcript levels of *HAS 2* gene were higher in E AD (*p* = 0.044) than in the other groups in the follicular phase, but not in the mid-luteal phase. Additionally, in all endometrosis groups, no differences in the mRNA levels of *HAS 2* were found between estrous cycle phases (Figure 7B).

## 4. Discussion

An interesting finding about the present results is the heterogeneity of gene transcription within a group. This can possibly indicate the complexity of endometrosis pathology and the involvement of some other pathways in cooperation with NF-κB. Additionally, despite showing similar histopathological features, some mares could have been in a different stage of the disease. Thus, allocation of endometrial samples to the specific classification groups of Hoffmann and co-authors (2009) could be somehow inaccurate. In a single endometrial biopsy, usually endometrial glands may show different types of cells. Therefore, a biopsy assignment to Hoffmann et al.’s classification groups has been made based on the state of most glands [1].

**Figure 8:**
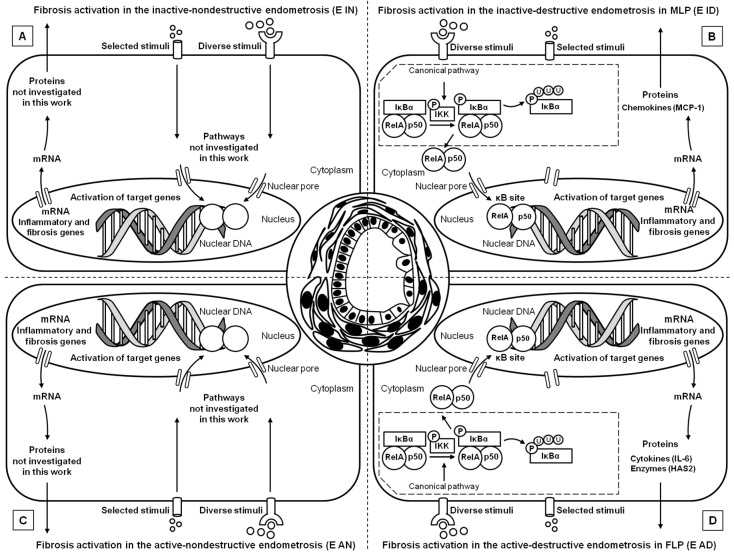
Schematic view of the NF-κB signaling pathway in (**A**) the inactive nondestructive endometrosis, (**B**) the inactive destructive endometrosis, (**C**) the active nondestructive endometrosis, and (**D**) the active destructive endometrosis. Adaptation of the whole NF-κB signaling pathway presented in Figure 1 in Domino et al. [20].

In a previous study, we evaluated the transcription of NF-κB pathway genes regarding Kenney and Doig’s endometrial categories, but not the histopathological types of endometrosis [20]. Interestingly, in the present study, the destructive type of endometrosis showed the highest differences in the transcription of several genes. In other studies, this type of endometrosis depicted a larger modification of ECM, especially the increase in proteoglycans, fibronectin and laminin expression [1,3]. Our results suggest that in this specific type of endometrosis, severe changes in ECM may be associated with the NF-κB pathway (Figure 8), which may regulate the production of connective tissue fibers. Since the destructive endometrosis is thought to decrease fertility more significantly than the non-destructive endometrosis, the NF-κB pathway might be involved in endometrial changes, which might impair pregnancy success [3].

Regarding Kenney and Doig´s categories, our previous study showed an increase in NF-κB gene transcripts in the canonical pathway activation (*NF-κB1*, *RelA*) and in the non-canonical pathway (*NF-κB2*), observed only in FLP along with the increase in endometrosis severity [20]. However, considering the various histopathological types of mare endometrosis, the main differences regard the canonical pathway of activation, since *RelA*, *NF-κB1*, and *MCP-1* genes transcripts increased in over than half of the endometria. It has been discovered that this pathway is coactivated by TNFα, another cytokine taking part in endometrosis pathogenesis, inducing fibroblasts’ transformation into myofibroblasts [21,32]. The known increased presence of TNFα in the endometrium may be responsible for the increase in NF-κB pathway proteins expression. The abovementioned activation may result in the promotion of MCP-1 expression, a potent factor increasing monocyte infiltration. Interestingly, these cells are the main producers of TNFα [32], thus this mechanism may act in a virtuous cycle.

Another explanation may be the fact that destructive endometrosis affects glands and glandular epithelium far more than nondestructive endometrosis [1,3]. The obtained results may show the influence of NF-κB on the glandular epithelium, causing its degeneration, alteration in secretion composition and basal lamina degradation, which altogether decreases fertility [8]. However, there is no direct evidence proving NF-κB involvement in basal lamina degradation, which needs to be further studied.

An increase in *RelA* transcription was noted in E AD in FLP and in E ID samples in MLP, similarly to *NF-κB1*, associated with the canonical pathway. In addition, *MCP-1* increased in E ID in MLP, whereas *IL-6* raised in E AD in FLP, thus showing that the transcription of those genes depended not only on the type of endometrosis, but also on the phase of the estrous cycle. In contrast, when considering Kenney and Doig’s endometrial classification, *IL-6* decreased significantly in FLP in samples with endometrosis [20]. These findings imply that endometrial changes induced by the NF-κB pathway are estrous cycle-dependent. Moreover, this may suggest that the metabolic activity of fibroblasts in the endometria of mares may depend on estrous cycle regulation and variability in ovarian steroid hormones levels. However, further studies, including estrogen and progesterone receptors evaluation, are needed to confirm this theory, considering the expression of proven receptors during endometrosis [1,2,3].

Among the hyaluronan synthases, only the transcription of *HAS 2* showed significant changes in the endometrosis samples evaluated in the present study. An increase in *HAS 2* mRNA levels in E AD tissue retrieved in the FLP, similarly to *RelA* transcript data, suggests a connection between these proteins in endometrosis pathogenesis. On the contrary, when considering Kenney and Doig´s categories, *HAS 3* gene transcriptions increased instead, in endometrosis tissues obtained in FLP [20]. Ohkawa et al. found that hyaluronan synthesis by fibroblasts is mediated by RelA, after stimulation by TNFα [33]. The difference found in gene transcription only in the follicular phase gives another evidence, that endometrosis pathogenesis might be somehow connected with the estrous cycle, as previously suggested [19].

The NF-κB is known for stimulating ECM deposition in various tissues [22,23,27,29,32]. A quantitative assessment of endometrosis can be carried out based on *RelA*, *NF-κB1*, and *MCP-1* gene transcription levels, as previously described for the use of uterocalin, uteroferrin, uteroglobin, and calbindin [8]. The achieved results may be helpful in the classification of endometrosis, as well as for the prognosis of disease development in clinical cases. However, further studies comparing NF-κB canonical pathway proteins and epithelial cell degeneration are necessary for confirmation of this assumption. This study has shown the importance of the NF-κB pathway on the pathogenesis of endometrosis. Since NF-κB inhibitors have been successfully studied in suppressing ECM deposition in various tissues [34,35,36], this approach should be further studied as a therapeutic means for endometrosis, allowing for stopping or even reversing fibrosis.

The main limitation of this study is that the obtained data only pertain to gene transcription, and the assessment of the end products of genes in the endometrium is necessary. The lack of immunohistochemistry for protein localization can be considered as a part of the explanation for the differing results of previous studies. Therefore, further studies encompass the use of immunohistochemistry for localization of proteins, and comparison among the various endometrosis types, which is crucial for further assumptions.

Summing up, activation of the NF-κB canonical pathway may be associated with degeneration and necrosis of glandular epithelial cells, as results showed significant changes in gene transcription in destructive endometrosis. Moreover, steroid hormones possibly modulate the NF-κB canonical pathway. Additionally, the activation of proinflammatory molecules, promoted by NF-κB, may play a role in gland deformation and damage, acting on residual inflammatory cells located in ECM, but also promoting infiltration of further leukocytes. In this study, *RelA*, *NF-κB2*, and *IL-6* transcription was increased in comparison with the control group in FLP in the active nondestructive type of endometrosis, whereas in our previous study, *RelA*, *NF-κB1*, *NF-κB2*, *HAS 1*, and *HAS 3* transcription similarly increased in FLP in the respective types of Kenney and Doig´s categories of endometrial classification when endometrosis was present [20]. Both findings suggest active remodeling of ECM in this phase of the cycle. Minor changes in the luteal phase may suggest that another set of chemokines present in the endometrium might be necessary to activate fibroblasts and myofibroblasts.

## 5. Conclusions

The NF-κB pathway is important in the regulation of fibrosis during endometrosis, regarding the different types of this condition, based on histopathological lesions. The NF-κB canonical pathway is upregulated especially in destructive fibrosis, indicating the highest intensity of changes in ECM deposition. The *MCP-1* gene transcription increased in the follicular phase in the inactive destructive type of endometrosis, whereas *IL-6* transcript levels raised in the mid-luteal phase in the active destructive endometrosis. Nevertheless, for further conclusions future studies are necessary, comprising more endometrial samples and additional research approaches.

## Data Availability

The data presented in this study are available on request from the corresponding author.

## Abbreviations

The following abbreviations are used in this manuscript:

C	endometrial samples classified to control group
E AD	endometrial samples classified to type active destructive
E AN	endometrial samples classified to type active nondestructive
E ID	endometrial samples classified to type inactive destructive
E IN	endometrial samples classified to type inactive nondestructive
ECM	extracellular matrix
FLP	follicular phases of the estrus
HAS 1	hyaluronan synthase 1
HAS 2	hyaluronan synthase 2
HAS 3	hyaluronan synthase 3
HE	hematoxylin-eosin staining
IL-6	interleukin-6
MCP-1	monocyte chemoattractant protein-1
MLP	mid-luteal phases of the estrus
NF-κB1	nuclear factor-κB subunit 1
NF-κB2	nuclear factor-κB subunit 2
qPCR	quantitative polymerase chain reaction
RelA	nuclear factor-κB subunit RelA
RT-PCR	real-time polymerase chain reaction
TNFα	tumor necrosis factor α

## References

[1] Hoffmann C., Ellenberger C., Mattos R.C., Aupperle H., Dhein S., Stief B., Schoon H.A. (2009). The equine endometrosis: New insights into the pathogenesis. Anim. Reprod. Sci..

[2] Schöniger S., Schoon H.A. (2020). The healthy and diseased equine endometrium: A review of morphological features and molecular analyses. Animals.

[3] Kenney R.M., Doig P.A., Morrow D.A. (1986). Equine endometrial biopsy. Current Therapy in Theriogenology.

[4] Lehmann J., Ellenberger C., Hoffmann C., Bazer F.W., Klug J., Allen W.R., Sieme H., Schoon H.A. (2011). Morpho-functional studies regarding the fertility prognosis of mares suffering from equine endometrosis. Theriogenology.

[5] Aupperle H., Schoon D., Schoon H.A. (2004). Physiological and pathological expression of intermediate filaments in the equine endometrium. Res. Vet. Sci..

[6] Bischofberger L., Szewczyk K., Schoon H.A. (2019). Unequal glandular dierentiation of the equine endometrium—A separate endometrial alteration?. Pferdeheilkunde.

[7] Minkwitz C., Schoon H.A., Zhang Q., Schöniger S. (2019). Plasticity of endometrial epithelial and stromal cells-A new approach towards the pathogenesis of equine endometrosis. Reprod. Dom. Anim..

[8] Hoffmann C., Bazer F.W., Klug J., Aupperle H., Ellenberger C., Schoon H.A. (2009). Immunohistochemical and histochemical identification of proteins and carbohydrates in the equine endometrium. Expression patterns for mares suffering from endometrosis. Theriogenology.

[9] Stewart F., Gerstenberg C., Suire S., Allen W.R. (2000). Immunolocalization of a novel protein (P19) in the endometrium of fertile and subfertile mares. J. Reprod. Fertil. Suppl..

[10] Schöniger S., Böttcher D., Theuß T., Gräfe H., Schoon H.A. (2016). New insights into the innate immune defences of the equine endometrium: In situ and in vitro expression pattern of beta-defensin. Pferdeheilkunde.

[11] Schöniger S., Gräfe H., Richter F., Schoon H.A. (2018). Expression of indoleamine 2,3-dioxygenase 1 as transcript and protein in the healthy and diseased equine endometrium. Res. Vet. Sci..

[12] Schöniger S., Gräfe H., Schoon H.A. (2018). Innate immunity mechanisms of the equine endometrium—Benefit or harm?. Pferdeheilkunde.

[13] Fouladi-Nashta A.A., Raheem K.A., Marei W.F., Ghafari F., Hartshorne G.M. (2017). Regulation and roles of the hyaluronan system in mammalian reproduction. Reproduction.

[14] Itano N., Sawai T., Yoshida M., Lenas P., Yamada Y., Imagawa M., Shinomura T., Hamaguchi M., Yoshida Y., Ohnuki Y. (1999). Three isoforms of mammalian hyaluronan synthases have distinct enzymatic properties. J. Biol. Chem..

[15] Stern R. (2004). Hyaluronan catabolism: A new metabolic pathway. Eur. J. Cell. Biol..

[16] Walter I., Handler J., Miller I., Aurich C. (2005). Matrix metalloproteinase 2 (MMP-2) and tissue transglutaminase (TG 2) are expressed in periglandular fibrosis in horse mares with endometrosis. Histol. Histopathol..

[17] Rodriguez H.I., Stewart A.J., Wolfe D.F., Caldwell F.J., Harrie M., Whitley E.M. (2011). Immunolocalization of the hyaluronan receptor CD44 in the reproductive tract of the mare. Theriogenology.

[18] Rebordão M.R., Galvao A., Szostek A., Amaral A., Mateus L., Skarzynski D.J., Ferreira-Dias G. (2014). Physiopathologic mechanisms involved in mare endometrosis. Reprod. Domest. Anim..

[19] Rebordão M.R., Amaral A., Lukasik K., Szóstek-Mioduchowska A., Pinto-Bravo P., Galvão A., Skarzynski D.J., Ferreira-Dias G. (2018). Constituents of neutrophil extracellular traps induce in vitro collagen formation in mare endometrium. Theriogenology.

[20] Domino M., Jasinski T., Kautz E., Juszczuk-Kubiak E., Ferreira-Dias G., Zabielski R., Sady M., Gajewski Z. (2020). Expression of genes involved in the NF-κB-dependent pathway of the fibrosis in the mare endometrium. Theriogenology.

[21] Szóstek-Mioduchowska A., Słowińska M., Pacewicz J., Skarzynski D.J., Okuda K. (2020). Matrix metallopeptidase expression and modulation by transforming growth factor-β1 in equine endometrosis. Sci. Rep..

[22] Ahn B.N., Song M.H., Kim J.H., Kim K.H., Park K.K., Choi Y.S. (2012). Intra-peritoneal NF-kappaB decoy oligodeoxynucleotide decreases postoperative intra-abdominal adhesion. Korean J. Obstet. Gynecol..

[23] Sosińska P., Baum E., Maćkowiak B., Staniszewski R., Jasinski T., Umezawa K., Bręborowicz A. (2016). Inhibition of NF-kappaB with Dehydroxymethylepoxyquinomicin modifies the function of human peritoneal mesothelial cells. Am. J. Transl. Res..

[24] Alekseevna R.V., Pavlovich D.A., Evgenievich B.Y., Viktorovich N.S. (2017). Nuclear factor kappa B as a potential target for pharmacological correction endothelium-associated pathology. Res. Res. Pharm..

[25] Arjmand M.H. (2020). The association between visceral adiposity with systemic inflammation, oxidative stress, and risk of post-surgical adhesion. Arch. Physiol. Biochem..

[26] Dejban P., Nikravangolsefid N., Chamanara M., Dehpour A., Rashidian A. (2021). The role of medicinal products in the treatment of inflammatory bowel diseases (IBD) through inhibition of TLR4/NF-kappaB pathway. Phytother. Res..

[27] May M.J., Ghosh S. (1998). Signal transduction through NF-κB. Trends Immunol..

[28] Lind D.S., Hochwald S.N., Malaty J., Rekkas S., Hebig P., Mishra G., Moldawer L.L., Copeland E.M., MacKay S. (2001). Nuclear factor-κB is upregulated in colorectal cancer. Surgery.

[29] Umezawa K. (2011). Possible role of peritoneal NF-κB in peripheral inflammation and cancer: Lessons from the inhibitor DHMEQ. Biomed. Pharmacother..

[30] Roberto da Costa R.P., Serrao P.M., Monteiro S., Pessa P., Robalo Silva J., Ferreira-Dias G. (2007). Caspase-3 mediated apoptosis and cell proliferation in the equine endometrium during the oestrous cycle. Reprod. Fertil. Dev..

[31] Schoon H.A., Schoon D., Klug E. (1997). Die Endometriumbiopsie bei der Stute im klinisch-gynäkologischen Kontext. Pferdeheilkunde.

[32] Brasier A.R. (2010). The nuclear factor-κB–interleukin-6 signalling pathway mediating vascular inflammation. Cardiovasc. Res..

[33] Ohkawa T., Ueki N., Taguchi T., Shindo Y., Adachi M., Amuroa Y., Hada T., Higashino K. (1999). Stimulation of hyaluronan synthesis by tumor necrosis factor-α is mediated by the p50/p65 NF–κB complex in MRC-5 myofibroblasts. Biochim. Biophys. Acta Mol. Cell Res..

[34] Caon I., Bartolini B., Moretto P., Parnigoni A., Carava E., Vitale D.L., Alaniz L., Viola M., Karousou E., De Luca G. (2017). Sirtuin 1 reduces hyaluronan synthase 2 expression by inhibiting nuclear translocation of NF-κB and expression of the long-noncoding RNA HAS2–AS1. J. Biol. Chem..

[35] Tong W., Geng Y., Huang Y., Shi Y., Xiang S., Zhang N., Qin L., Shi Q., Chen Q., Dai K. (2015). In vivo identification and induction of articular cartilage stem cells by inhibiting NF-κB signaling in osteoarthritis. Stem Cells.

[36] Chung S., Son M., Kim M., Koh E.S., Shin S.J., Park C.W., Kim S., Kim H.S. (2019). Inhibition of p300/CBP-associated factor attenuates renal tubulointerstitial fibrosis through modulation of NF-kB and Nrf2. Int. J. Mol. Sci..

